# Supplementary material for: High PD-L1 Expression in HRS Cells and Macrophages in Tumor Immune Microenvironment Is Associated with Adverse Outcome and EBV Positivity in Classical Hodgkin Lymphoma
Source: Int J Mol Sci. 2025 Jun 11;26(12):5592. doi: 10.3390/ijms26125592 (PMC12192575; doi:10.3390/ijms26125592)
Supplement: Supplementary file 1 [file ijms-26-05592-s001.zip › Table S1 & S2.pdf]

Table S1. Modelling hazard* of dying with PDL-1 expression on macrophages and HRS cells along with the classical clinical pathological variables

Variable	β	85% CI of β	P value
Intercept	-3.36	-4.678 to -2.051	0.0004
age (< or >45 y.)#	0.01	-0.5655 to 0.5851	0.9803
clinical stage	0.91	0.5122 to 1.301	0.0013
hist. subtype (NS 1, MC 2, LR 3, LD 4)	0.19	-0.2604 to 0.6373	0.5433
extranodal disease#	-1.58	-2.266 to -0.8897	0.0013
EBV*	0.29	-0.3625 to 0.9499	0.5172
PDL1 HRS (H score)#	*0.0002*	*-0.5232 to 0.5237*	*0.9994*
%PDL1+ MΦ#	*0.35*	*-0.1810 to 0.8843*	*0.3401*
OS time (year)	-0.12	-0.1799 to -0.06426	0.003
R^2=30.02%, AICc=88.89			
Intercept	-3.02	-4.122 to -1.917	0.0001
age (< or >45 y.)#	0.08793	-0.4698 to 0.6457	0.8193
clinical stage	0.8701	0.4834 to 1.257	0.0016
hist. subtype (NS 1, MC 2, LR 3, LD 4)	0.1294	-0.3057 to 0.5645	0.6668
extranodal disease#	-1.52	-2.197 to -0.8428	0.0016
EBV#	0.3653	-0.2411 to 0.9718	0.3839
OS time (year)	-0.1308	-0.1851 to -0.07649	0.0008
R^2=29.19%, AICc=84.96			

*Logit model was used i.e. $logit(h) = \beta_0 + \beta_1 \cdot x_1 + \cdots + \varepsilon$;# Binary variables

Table S2. Modelling hazard* of relapse with PDL-1 expression on macrophages and HRS cells along with the classical clinical pathological variables

Variable	beta estimate	85% CI	P value
Intercept	-0.03167	-1.057 to 0.9934	0.9643
age (< or >45 y.)#	-0.2044	-0.6530 to 0.2442	0.5096
clinical stage	0.4847	0.1805 to 0.7890	0.0232
hist. subtype (NS 1, MC 2, LR 3, LD 4)	0.3433	0.004227 to 0.6824	0.1451
extranodal disease#	-0.401	-0.9224 to 0.1204	0.2669
EBV#	0.09112	-0.4013 to 0.5836	0.7886
PDL1 HRS#	0.4158	0.01113 to 0.8204	0.1393
%PDL1+ MΦ#	0.2529	-0.1472 to 0.6531	0.3609
PFS time (year)	-0.9314	-1.071 to -0.7921	<0.0001
PFS time^2 (year^2)	0.04393	0.03470 to 0.05315	<0.0001
R^2=72.99%, AICc=39.36			
Intercept	0.4594	-0.4495 to 1.368	0.4646
age (< or >45 y.)#	-0.2228	-0.6606 to 0.2151	0.4617
clinical stage	0.446	0.1437 to 0.7482	0.0351
hist. subtype (NS 1, MC 2, LR 3, LD 4)	0.2901	-0.04376 to 0.6240	0.2102
extranodal disease#	-0.3455	-0.8641 to 0.1732	0.3358
EBV#	0.3253	-0.1271 to 0.7777	0.2991
PFS time (year)	-0.9196	-1.057 to -0.7823	<0.0001
PFS time^2 (year^2)	0.04215	0.03312 to 0.05119	<0.0001
R^2=71.96%, AICc=37.49			

*Logit model was used i.e. $logit(h) = \beta_0 + \beta_1 \cdot x_1 + \cdots + \varepsilon$; # Binary variables
