# Supplementary figures and images for: High PD-L1 Expression in HRS Cells and Macrophages in Tumor Immune Microenvironment Is Associated with Adverse Outcome and EBV Positivity in Classical Hodgkin Lymphoma
Source: Int J Mol Sci. 2025 Jun 11;26(12):5592. doi: 10.3390/ijms26125592 (PMC12192575; doi:10.3390/ijms26125592)

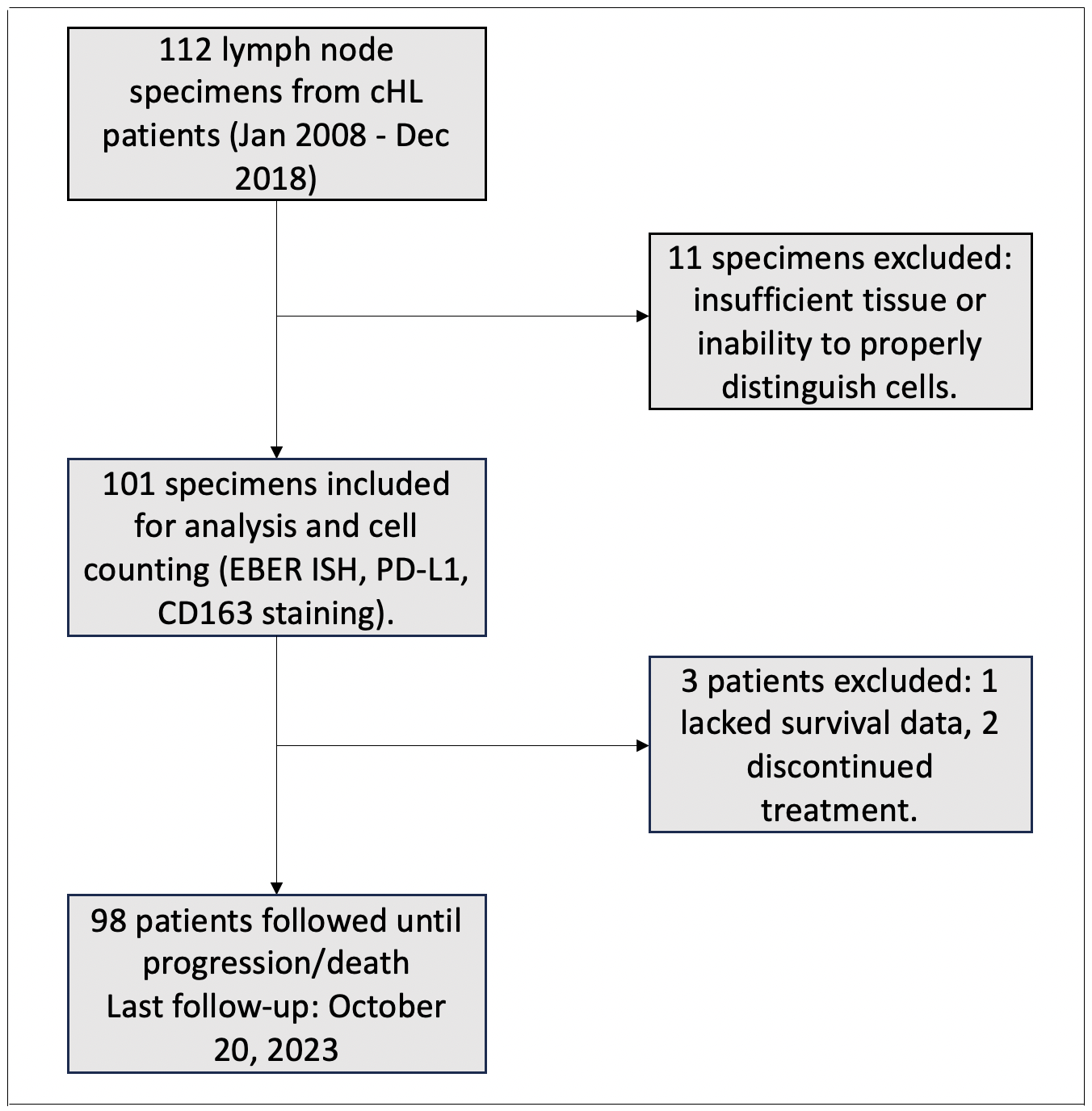

Supplement: Supplementary file 1 [file ijms-26-05592-s001.zip › Figure S1.png]
